# Residual force enhancement is not altered while force depression is amplified at the cellular level in old age

**DOI:** 10.1242/jeb.248155

**Published:** 2025-01-13

**Authors:** Binta S. Njai, Avery Hinks, Makenna A. Patterson, Geoffrey A. Power

**Affiliations:** Department of Human Health and Nutritional Sciences, College of Biological Sciences, University of Guelph, Guelph, ON, Canada, N1G 2W1

**Keywords:** Isometric force, Muscle architecture, Stiffness, Cross-bridge, History dependence of force, Single fibre

## Abstract

Residual force enhancement (rFE) and residual force depression (rFD) are history-dependent properties of muscle which refer to increased and decreased isometric force following a lengthening or shortening contraction, respectively. The history dependence of force is greater in older than in younger human adults when assessed at the joint level. However, it is unclear whether this amplification of the history dependence of force in old age is owing to cellular mechanisms or is a consequence of age-related remodelling of muscle architecture. Single muscle fibres from the psoas major of old and young F344BN rats were dissected and chemically permeabilized. Single muscle fibres were mounted between a force transducer and length controller, then maximally activated (pCa 4.5). To assess rFD, fibres were actively shortened from 3.1 to 2.5 µm at both a slow (0.15 *L*_o_ s^−1^) and fast (0.6 *L*_o_ s^−1^) speed, with a fixed-end isometric reference contraction at 2.5 µm. To assess rFE, fibres were activated and stretched at 0.3 *L*_o_ s^−1^ from a sarcomere length of 2.2 to 2.5 µm, and 2.7 to 3.0 µm, and compared with fixed-end isometric reference contractions at 2.5 and 3.0 µm, respectively. Isometric force (2.5 µm) was ∼19% lower in muscle fibres from old as compared with young rats (*P*<0.001). Upon normalizing to fibre cross-sectional area, there was no age-related difference in specific force (*P*>0.05). rFD was ∼33% greater in muscle fibres from old as compared with young rats (*P*<0.05), while rFE did not differ between groups (*P*>0.05). rFD is amplified in old age at the cellular level, while rFE appears to be unchanged; thus, previously reported age-related modification of rFE occurs upstream from the cellular level.

## Teaser

**Summary:** Residual force depression is amplified in old age at the cellular level, while residual force enhancement appears to be unchanged; thus, previously reported age-related modification of rFE likely occurs upstream of the cellular level.

## INTRODUCTION

The history-dependent properties of muscle known as residual force enhancement (rFE) and residual force depression (rFD) refer to an increase and decrease in isometric force following a lengthening (i.e. eccentric) or shortening (i.e. concentric) contraction, respectively, as compared with a fixed-end isometric contraction at the same muscle length and level of activation (Abbott and Aubert, 1952; Hahn et al., 2023; Seiberl et al., 2015). The presence of rFE and rFD has been observed in many species across various scales of muscle (Chen et al., 2020; Joumaa et al., 2008; Leonard et al., 2010; Mashouri et al., 2021; Pinnell et al., 2019; Ramsey et al., 2010), and at the joint level in humans during electrically stimulated and voluntary contractions (Chapman et al., 2018; Chen et al., 2019; Hahn et al., 2023; Seiberl et al., 2015). Both rFE and rFD appear to be greater in older as compared with younger humans when assessed at the joint level during voluntary contractions (Power et al., 2012, 2014a, 2015). However, it is unclear whether this amplification of the history dependence of force in old age is owing to cellular mechanisms or a consequence of age-related muscle architecture remodelling.

The mechanisms of rFE and rFD originate at the cellular level. For rFE, in the presence of Ca^2+^ and cross-bridge cycling, ‘the molecular spring’ titin becomes stiffer, thereby contributing greater tension during and following active lengthening of the muscle (Hahn et al., 2023; Herzog, 2018; Hessel et al., 2024), resulting in a greater contribution of passive force to total force production as compared with a fixed-end isometric contraction (Herzog and Leonard, 2002). As well, rFE is greatest following large stretch amplitudes to long muscle lengths (Bakenecker et al., 2022; Fukutani et al., 2017; Rassier and Herzog, 2004). rFD occurs as a consequence of an inhibition of cross-bridge attachment following active shortening of the muscle, limiting available binding sites, resulting in fewer attached force-generating cross-bridges as compared with a fixed-end isometric contraction (Hahn et al., 2023; Joumaa et al., 2017, 2021), and is proportional to work of shortening (Herzog et al., 2000).

Interestingly, both rFE and rFD were amplified in older compared with young adult humans at the joint level (Power et al., 2012, 2014a, 2015). This age-related amplification of the history dependence of force may be explained by changes in muscle architecture, as muscle fascicle length decreases with age (Hinks et al., 2024a; Narici et al., 2003; Power et al., 2013a, 2021). Therefore, older individuals with shorter muscle fascicles may have experienced relatively greater changes in sarcomere length during joint-level active lengthening and shortening contractions, leading to greater rFE and rFD, respectively. Whether the amplification of the history dependence of force in old age originates at the cellular level has not been investigated, leaving the mechanisms behind an amplification of rFE and rFD in old age unclear. Therefore, in the present study, we investigated whether age-related differences in rFE and rFD exist at the single muscle fibre level in male F344BN F1 rats.

## MATERIALS AND METHODS

### Animals

Ten young (age at euthanasia ∼8 months; mean±s.d. mass 381.61±15.71 g) and 14 old (age at euthanasia ∼32 months; mass 480.79±37.91 g) male F344BN F1 rats were obtained from the National Institute on Aging aged rodent colonies (Charles River Laboratories, Senneville, QC, Canada) with approval from the Animal Care Committee (AUP: #4905) at the University of Guelph, and all protocols followed guidelines from the Canadian Council on Animal Care. Rats were housed in groups of two or three with a 12 h:12 h light:dark cycle at 23°C and were given unrestricted access to room-temperature water and a Teklad global 18% protein rodent diet (Envigo, Huntington, Cambridgeshire, UK). Rats were killed via isoflurane followed by CO_2_ asphyxiation then cervical dislocation prior to harvesting muscle tissue. All procedures were approved by the Animal care committee of the University of Guelph (AUP: #4905).

### Tissue preparation

The right and left psoas major muscles were harvested from the rats and immediately transferred to a silicone elastomer-plated Petri dish containing chilled dissecting solution. The psoas major muscle was selected owing to its long fibres and almost complete fast-type fibre composition (Hämäläinen and Pette, 1993; Schilling et al., 2005; Vlahovic et al., 2017). Fibre bundles were dissected from the proximal half of the psoas major, which is almost exclusively fast-type fibres (<1% type I) (Hämäläinen and Pette, 1993). The muscles were then sectioned into fibre bundles and transferred to a tube containing 2.5 ml of chilled skinning solution, where they remained on ice for 30 min for permeabilization (Hubbard et al., 2023; Mazara et al., 2021). The bundles were then washed with fresh chilled dissecting solution and gently agitated to ensure any remaining skinning solution was removed. The bundles were then transferred to a tube containing storage solution and incubated for 24 h at 4°C. Tubes were prepared with fresh storage solution and the bundles were placed in individual tubes and stored in a freezer at −80°C until mechanical testing began (Hubbard et al., 2023; Mashouri et al., 2021), as is advised by the protocol of Roche and colleagues (2015). Any remaining fibres at the end of the testing day were discarded and not refrozen.

### Solutions

The dissecting solution was composed (mmol l^−1^): K-propionate (250), imidazole (40), EGTA (10), MgCl_2_·6H_2_O (4), Na_2_H_2_ATP (2). The storage solution was composed of (mmol l^−1^): K-propionate (250), imidazole (40), EGTA (10), MgCl_2_·6H_2_O (4), Na_2_H_2_ATP (2), glycerol (50% of total volume after transfer to 50:50 dissecting:glycerol solution). The skinning solution with Brij 58 was composed of (mmol l^−1^): K-propionate (250), imidazole (40), EGTA (10), MgCl_2_·6H_2_O (4), 1 g of Brij 58 (0.5% w/v). The relaxing solution was composed of (mmol l^−1^): imidazole (59.4), K-methanesulfonic acid (MSA) (86), Ca(MSA)_2_ (0.13), Mg(MSA)_2_ (10.8), K_3_EGTA (5.5), KH_2_PO_4_ (1), H_2_O, leupeptin (0.05), Na_2_ATP (5.1). The pre-activating solution was composed of (mmol l^−1^): K-propionate (185), Mops (20), Mg(CH_3_COO)_2_ (2.5), Na_2_ATP (2.5). The activating solution (pCa 4.5) was composed of (mmol l^−1^): Ca^2+^ (15.11), Mg (6.93), EGTA (15), Mops (80), Na_2_ATP (5), CP (15). All solutions were adjusted to a pH of 7.0 with the appropriate acid (HCl) or base (KOH).

### Mechanical testing and force measurements

On testing days, single muscle fibres were isolated from a fibre bundle and moved to a temperature-controlled chamber filled with relaxing solution where they were then tied to pins connected to a force transducer (403A, Aurora Scientific, Toronto, ON, Canada) and a length controller (322C, Aurora Scientific) with nylon sutures. All experiments were performed at 12°C to allow for a balance between force production capacity and the number of contractions performed before experiencing force loss (Bottinelli et al., 1996; Ranatunga, 1982, 1984; Tomalka et al., 2017, 2021). To ensure fibre viability throughout testing, it is important to note that our testing was conducted at 12°C; therefore, our results may not entirely mimic what occurs in physiological temperature conditions. Muscle contraction was initiated by first transferring the fibre from relaxing solution to a pre-activating solution with reduced Ca^2+^ buffering capacity for 10 s before being transferred to an activating solution. All activations were performed in a maximal Ca^2+^ concentration solution (pCa 4.5). For all tests, the average sarcomere length (SL) was measured using a high-speed camera (Aurora Scientific Inc., HVSL 901A). Total force was recorded, and active force was determined by subtracting passive from total force. Before starting the testing protocol, the fibre was set to a SL of 2.5 μm (i.e. the optimal SL for rat muscle fibres; Burkholder and Lieber, 2001; Ledvina and Segal, 1995) and a ‘fitness’ contraction was performed to ensure the ties were not loose and the condition of the fibre was sufficient for testing. After the fitness test, SL was re-measured and, if necessary, re-adjusted to 2.5 μm and passive force re-measured. Then, the cross-sectional area (CSA) of each fibre was determined. This was done by measuring the diameter of the fibre in three places (at each end and in the centre) using an eyepiece reticle. These three measurements were then averaged to calculate the CSA of the individual fibre. Given these samples are muscle fibre segments and chemically permeabilized, we treated them as cylindrical, and assumed circularity in the calculation of CSA.

### Residual force depression

**Fig. 1.**
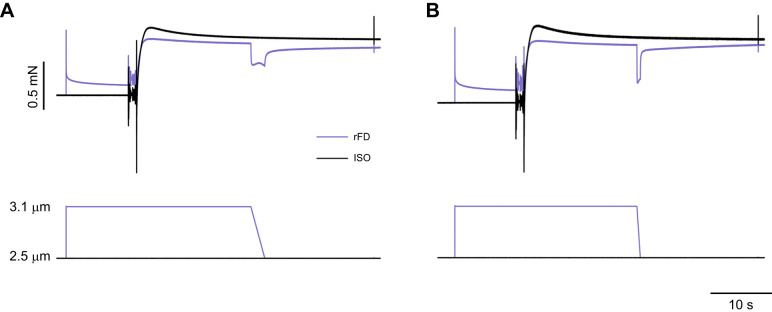
**Representative force traces for residual force depression (rFD) and isometric reference (ISO) conditions.** In the rFD protocol, single muscle fibres were passively lengthened to a sarcomere length (SL) of 3.1 µm, activated and shortened to 2.5 µm (i.e. plateau of the force–length relationship) at (A) a slow speed of 0.15 *L*_o_ s^−1^ (where *L*_o_ is the optimal length for force production) and (B) an increased speed of 0.6 *L*_o_ s^−1^. Representative force traces of a slow/fast velocity shortening contraction (purple) and isometric reference contraction (black) are shown. An instantaneous stiffness test was performed in both conditions prior to deactivation.

Fibres were set to an average SL of 2.5 μm, then passively lengthened to 3.1 μm. Activation began 10 s after lengthening to 3.1 µm. After 20 s of activation, fibres were actively shortened to an average SL of 2.5 μm at 0.15 *L*_o_ s^−1^ (slow speed, where *L*_o_ is optimal length; Fig. 1A) and held for 15 s to reach an isometric steady state before deactivation. The same protocol was repeated with a velocity of 0.6 *L*_o_ s^−1^ (fast speed; Fig. 1B), with the order randomized between the two velocities. For the isometric reference contraction (ISO), fibres were activated at an average SL of 2.5 μm for 35 s.

### Residual force enhancement

**Fig. 2.**
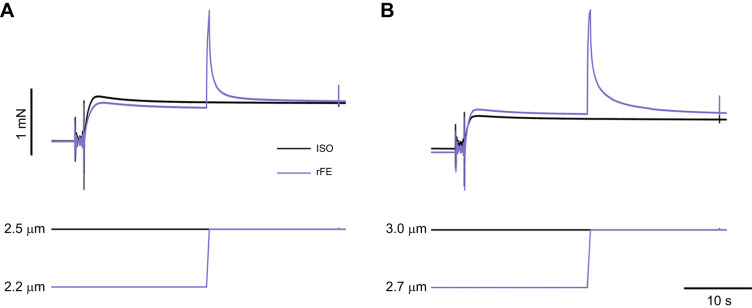
**Representative force traces for residual force enhancement (rFE) and isometric reference (ISO) conditions.** In the rFE protocol, single muscle fibres were (A) set to an average SL of 2.2 µm and passively stretched to 2.5 µm (i.e. plateau of the force–length relationship) at 0.3 *L*_o_ s^−1^ or (B) set to an average SL of 2.7 µm and passively stretched to 3.0 µm (i.e. descending limb of the force–length relationship) at 0.3 *L*_o_ s^−1^. Representative force traces for force enhancement (purple) and isometric reference (black) conditions for a young single fibre are shown.

For assessment of rFE near the plateau region of the force–length relationship, first, a reference isometric contraction was performed at a SL of 2.5 µm. For the stretch condition, fibres were set to an average SL of 2.5 µm and passively shortened to 2.2 µm, then 10 s following passive shortening, were activated. After 20 s of activation, fibres were actively stretched to 2.5 µm at 0.3 *L*_o_ s^−1^ and held for 15 s before deactivation (Fig. 2A). For assessment of rFE on the descending limb of the force–length relationship, a reference isometric contraction was performed at a SL of 3.0 µm. Fibres were set to an average SL of 3.0 µm and passively shortened to 2.7 µm. Activation began 10 s after passive shortening. After 20 s of activation, fibres were actively stretched to 3.0 µm at 0.3 *L*_o_ s^−1^ and held for 15 s to reach an isometric steady state before deactivation (Fig. 2B).

### Analysis

Based on previous protocols from our lab (Patterson et al., 2024), to ensure fibre fidelity, we excluded any fibres that produced isometric active specific force less than 20 mN mm^−2^. While this force value is on the low side for rat single fibres, the main outcomes were relative (%) rFE and rFD in which the reference isometric force is compared with isometric force following stretch or shortening and expressed as a relative change within each individual fibre. Furthermore, we removed any fibres from the analysis that experienced greater than a 20% drop in force from the first ISO contraction of the rFD protocols to the ISO contraction of the short rFE protocol (both were at a sarcomere length of 2.5 µm). Next, the force traces of any fibres that exhibited positive rFD (i.e. greater steady-state force in the rFD contraction compared with the ISO contraction) were manually inspected for any signs that the fibre ripped or there were other errors in the signal. If these issues were observed, those fibres were removed from analysis. We manually inspected the force traces of any fibres that exhibited negative rFE (i.e. lower steady-state force in the rFE contraction compared with the ISO contraction) for any signs of ripping or eccentric contraction-induced damage. If damage-induced force loss was evident, those fibres were removed from the analysis. Lastly, because more rFE than rFD protocols were removed from the initial analyses, additional fibres were tested for only the rFE protocols to ensure sample sizes among all protocols were balanced. Altogether, these data inclusion steps left us with a final sample size of 40 old and 49 young fibres for the fast rFD protocol, 42 old and 48 young fibres for the slow rFD protocol, 49 old and 54 young fibres for the short rFE protocol, and 43 old and 48 young fibres for the long rFE protocol. *N*=2–11 single fibres from each animal were included in the analysis, with each fibre considered an independent sample.

### Work of shortening

Mechanical work of shortening during the rFD protocols was calculated as the product of the area under the curve of the force–time trace during shortening and the change in fibre length.

### Stiffness

Instantaneous stiffness (*k*) tests were performed to determine the proportion of attached cross-bridges during the force plateau. This was done by rapidly stretching the fibre (500 *L*_o_ s^−1^) by 0.3% of *L*_o_ s^−1^ and dividing the change in force during the stretch by the change in length.

### History-dependent properties of force

For rFD, rFE and the ISO values, force was reported as an average over 500 ms before the instantaneous stiffness test at the same time following activation for all conditions (Figs 1 and 2). For the rFE and rFD trials, the 500 ms time point where average force was reported corresponded to the isometric steady-state 15 s following the dissipation of lengthening/shortening force transients. rFD was calculated as the difference between the average force in the rFD condition compared with the ISO condition at 2.5 μm. Similarly, stiffness depression was calculated as the percentage decrease in instantaneous stiffness in the rFD state compared with the ISO condition (Joumaa et al., 2012). rFE was calculated as the difference between the force in the rFE condition compared with the ISO condition at 2.5 μm (short) and 3.0 μm (long). rFD experiments were always performed first and randomized across speeds, with the shortening condition preceding the isometric reference contraction. rFE experiments were performed after the rFD contractions owing to their potential for muscle damage, with the short condition first followed by the long condition. The isometric reference contraction always preceded the corresponding rFE condition.

All statistical analyses were performed in SPSS Statistics Premium 28. Age-related differences in fibre CSA, maximum isometric force at 2.5 μm and instantaneous stiffness were assessed by one-way analysis of variance (ANOVA). Age- and speed-related differences in rFD, stiffness depression and work of shortening were assessed via two-way ANOVA [speed (fast, slow)×age (young, old)]. Age- and length-related differences in rFE were assessed by a two-way ANOVA [length (short, long)×age (young, old)]. To assess the relationship between percentage rFD and percentage stiffness depression, a linear regression was performed between percentage rFD and the change in instantaneous stiffness between the ISO and the history-dependent conditions. Data are reported in figures as means±s.d., α=0.05.

## RESULTS

**Fig. 3.**
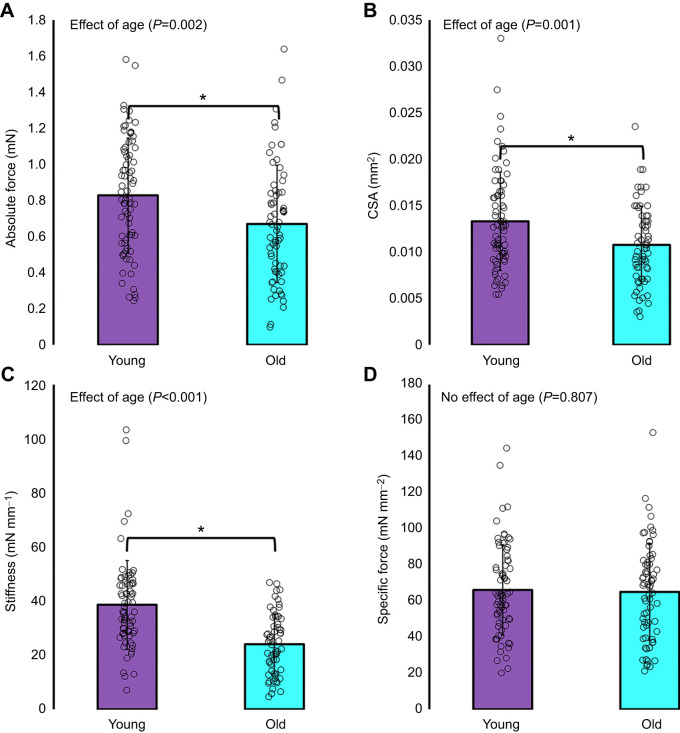
**Single muscle fibre cross-sectional area (CSA) and isometric force properties.** Absolute force measured at 2.5 µm (*n*=75 young, *n*=69 old) (A), cross-sectional area (CSA; *n*=75 young, *n*=69 old) (B) and instantaneous stiffness (*n*=72 young, *n*=65 old) (C) were lower in single muscle fibres from old as compared with young rats. When force was normalized to CSA, there was no age-related difference in specific force (*n*=75 young, *n*=69 old) (D). *Significant difference between young and old (*P*<0.05).

Single muscle fibres from old rats were ∼19% weaker (*F*_1144_=9.589, *P*=0.002; Fig. 3A), with an ∼19% smaller CSA (*F*_1144_=11.257, *P*=0.001; Fig. 3B) and ∼48% lower instantaneous stiffness (*F*_1137_=36.811, *P*<0.001) at maximal activation (i.e. a smaller proportion of attached force-producing cross-bridges in old fibres; Fig. 3C) as compared with those from young rats. When force was normalized to CSA to account for the smaller fibres in old rats, there was no age-related difference (*F*_1144_=0.060, *P*=0.807) in specific force (Fig. 3D), indicating intrinsic muscle quality was intact.

### Work of shortening

**Fig. 4.**
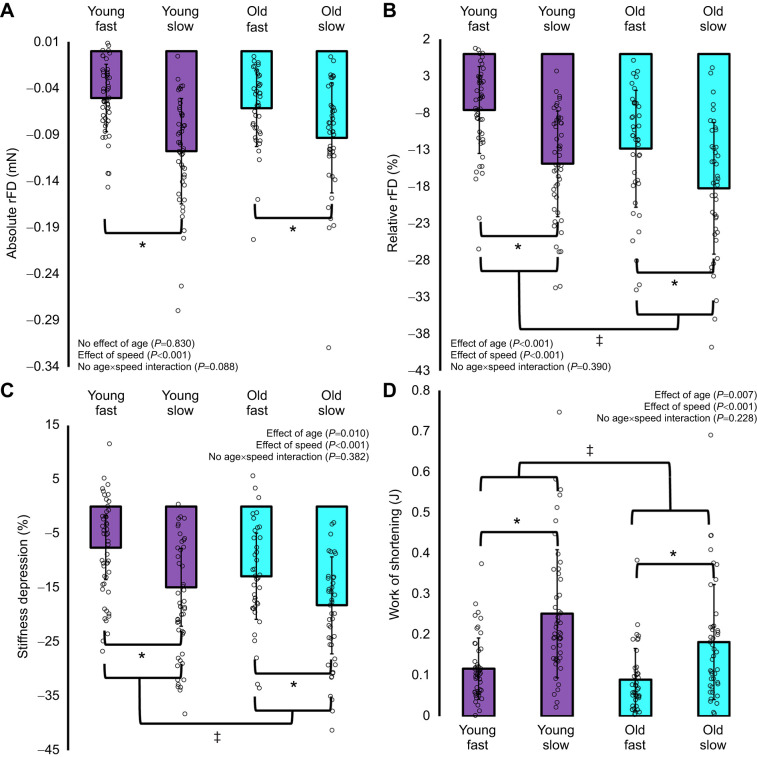
**Residual force depression (rFD), stiffness and work of shortening.** Absolute (A) and relative (B) rFD (rFD fast: *n*=49 young, *n*=40 old; rFD slow: *n*=48 young, *n*=42 old), percentage stiffness depression (C) (rFD fast: *n*=49 young, *n*=40 old; rFD slow: *n*=48 young, *n*=42 old) and work (D) (rFD fast: *n*=49 young, *n*=40 old; rFD slow: *n*=48 young, *n*=42 old) in young and old fibres when shortening from an average sarcomere length of 3.1 to 2.5 µm at a fast (0.60 *L*_o_ s^−1^) and slow speed (0.15 *L*_o_ s^−1^). Both absolute and relative rFD were greater in the slow than fast condition, and old fibres experienced greater rFD (%) as compared with young fibres. There was a greater reduction in stiffness in the slow speed condition, and old fibres had greater stiffness depression as compared with young fibres. Work was greater during the slow as compared with fast condition, and lower in old as compared with young fibres. *Significant difference between slow and fast with young and old combined (*P*<0.05). ^‡^Significant difference between young and old with slow and fast combined (*P*<0.05).

For work performed during the active shortening contractions, there was no interaction of age×speed (*F*_1179_=1.462, *P*=0.228), but there were main effects for both age (*F*_1179_=7.385, *P*=0.007) and speed of shortening (*F*_1179_=40.881, *P*<0.001), such that old fibres performed ∼26% less work than young fibres, and ∼110% more work was performed during the slow as compared with the fast speed condition (Fig. 4D).

### Residual force depression

For absolute rFD (mN), there was no interaction of age×speed (*F*_1179_=2.947, *P*=0.088), nor an effect of age (*F*_1179_=0.046, *P*=0.830) but, as expected, rFD was ∼80% greater for the slow as compared with fast speed condition (*F*_1179_=36.469, *P*<0.001; Fig. 4A), owing to greater work of shortening during the slow as compared with the fast condition. For relative rFD (%), there was no interaction of age×speed (*F*_1179_=0.742, *P*=0.390); however, there were main effects for both age (*F*_1179_=14.664, *P*<0.001) and speed (*F*_1179_=31.883, *P*<0.001), such that old fibres had ∼38% greater rFD as compared with young fibres despite performing less mechanical work of shortening, and there was ∼62% more rFD for the slow as compared with the fast condition (Fig. 4B).

### Stiffness depression

For instantaneous stiffness depression (%), there was no interaction of age×speed (*F*_1179_=0.768, *P*=0.382), but there was an effect of speed, with ∼62% greater reduction in stiffness for the slow as compared with the fast condition (*F*_1179_=36.616, *P*<0.001). As well, there was ∼38% greater stiffness depression in old compared with young fibres (*F*_1179_=6.787, *P*=0.010; Fig. 4C); notably, this was the same magnitude of age-related difference as for relative rFD.

### Linear regression analysis

**Fig. 5.**
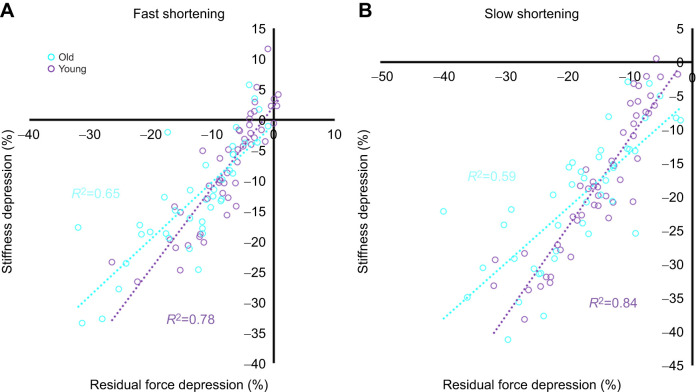
**Linear regression analysis of stiffness depression versus rFD for the slow and fast condition in young and old fibres.** (A) Fast condition: young group (purple dotted line, *R*^2^=0.78, *P*<0.05), old group (blue dotted line, *R*^2^=0.65, *P*<0.05). (B) Slow condition: young group (purple dotted line, *R*^2^=0.84, *P*<0.05), old group (blue dotted line, *R*^2^=0.59, *P*<0.05). rFD fast: *n*=49 young, *n*=40 old; rFD slow: *n*=48 young, *n*=42 old.

For both the fast (Fig. 5A) and slow (Fig. 5B) shortening speed conditions, in both young and old fibres there were strong relationships (*R*^2^=0.59–0.84, all *P*<0.001) between rFD (%) and stiffness depression (%). These relationships tended to be slightly weaker in old (*R*^2^=0.59–0.65) as compared with young fibres (*R*^2^=0.78–0.84), such that stiffness depression did not explain as much of the variance in rFD in old as in young fibres, indicating that the greater rFD in old fibres may be driven by additional factors outside of fewer attached cross-bridges.

### Residual force enhancement

**Fig. 6.**
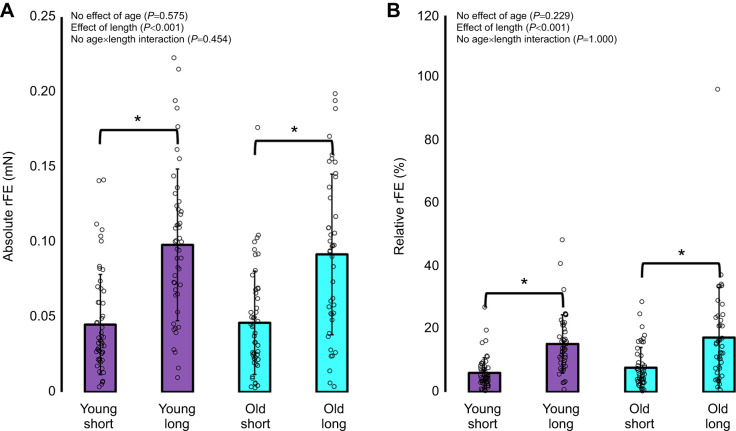
**Residual force enhancement (rFE).** Absolute (A) and relative (B) force enhancement on the plateau (short; sarcomere length: 2.5 µm) (*n*=54 young, *n*=49 old) and descending limb (long; sarcomere length: 3.0 µm) (*n*=48 young, *n*=43 old) of the force–length relationship. rFE was greater at longer as compared with shorter sarcomere lengths, with no age-related differences. *Difference between short and long with young and old combined (*P*<0.05).

For absolute rFE (mN), there was no interaction of age×length (*F*_1195_=0.562, *P*=0.454), nor an effect of age (*F*_1195_=0.315, *P*=0.575), but as expected, rFE was ∼109% greater on the descending limb (sarcomere length: 3.0 µm) as compared with the plateau region (sarcomere length: 2.5 µm) of the force–length relationship (*F*_1195_=59.968, *P*<0.001; Fig. 6A). For relative rFE (%), there was no interaction of age×length (*F*_1195_=0.000, *P*=1.00), nor an effect of age (*F*_1195_=1.456, *P*=0.229), and like absolute rFE, relative rFE was ∼135% greater on the descending limb (long) as compared with the plateau region (short) of the force–length relationship (*F*_1195_=42.950, *P*<0.001; Fig. 6B).

## DISCUSSION

The history dependence of force is an intrinsic property of muscle currently unexplained by the cross-bridge theory. In the present study, we show that the amplification of rFD in old rats originates at the cellular level and is indeed an intrinsic property of muscle which likely scales to joint-level voluntary contractions in humans. Meanwhile, the cellular mechanisms contributing to rFE in old age are not altered; therefore, previous reports of greater rFE in old age at the joint level in humans are likely driven by factors upstream of the single muscle fibre.

### Age-related impairments in force production

Single muscle fibres from old rats exhibited typical age-related declines in maximal isometric force production, CSA and instantaneous stiffness (Lim and Frontera, 2022, 2023; Mazara et al., 2021). However, intrinsic contractility of the muscle appears to be intact as specific force (i.e. muscle quality) was not different between old and young fibres when force was normalized to CSA, accounting for the age-related decrease in contractile tissue (Fig. 3). Across studies, age-related changes to specific force are equivocal; some report an increase with age, while others report no change or a decrease (Bruce et al., 1989; Larsson and Ansved, 1995; Larsson et al., 2019). In rodent muscles with a predominately Type II fibre distribution, such as the psoas major, some reported specific force decreases with age, although not to the extent of absolute force decreases (Larsson and Edström, 1986; Brooks and Faulkner, 1988; Degens et al., 1995; McBride et al., 1995; Wineinger et al., 1995; Lowe et al., 2001).

### History dependence of force

In the present study, our values of rFE at short and long lengths averaged 7% and 16%, respectively. As well, rFD values for the slow and fast shortening condition averaged −16% and −10%, respectively. These values are consistent with data published previously for single muscle fibres from the human vastus lateralis (rFD 0–19%, rFE 6–25%; Pinnell et al., 2019), and rabbit psoas and soleus (rFD 1–29%; Joumaa et al., 2015). Some of our rFD values were close to 0% or positive; our conservative approach in testing order may have contributed, whereby performing rFD first before the reference fixed-end isometric contraction could have lowered the force value recorded. However, consistent with any of the low rFD values is a similar lack of stiffness depression (Fig. 5), indicating that inhibition of cross-bridge attachment following active shortening did not occur in those fibres. While fibre type was not assessed, these values may represent some of the slowest fibres, unable to perform sufficient work of shortening during the fast condition, inducing negligible rFD. Although rFE appears to be velocity independent (Herzog, 2004), rFD is dependent on the work of shortening, and therefore is greater during slow contractile speeds where the muscle can generate higher forces than at fast speeds (Herzog, 2004). We observed this velocity dependence for rFD in the present study in single fibres from both young and old rats (Fig. 4). Muscle fibre type has been suggested to influence the history dependence of force, with fast-type muscle having greater magnitudes of rFE (Ramsey et al., 2010) and rFD (Joumaa et al., 2015). However, others have not observed a fibre-type dependence for rFE (Pinnell et al., 2019). Given rFD is dependent on work of shortening, fast-type muscles experience greater rFD for a given speed of shortening as compared with slow-type muscles. Upon normalizing to maximal shortening velocity, rFD does not appear to differ across fibre types (Joumaa et al., 2015). We tested muscle fibres from the proximal portion of the psoas major, which is composed almost exclusively of type II fibres (Hämäläinen and Pette, 1993; Schilling et al., 2005; Vlahovic et al., 2017). It is unlikely that any differences in fibre type between young and old rats influenced our findings, as ageing is typically associated with a greater proportion of slow-type fibres (Hepple and Rice, 2016; Kanda and Hashizume, 1989), and if old rats had more slow-type fibres in the present study, they would have likely experienced less (rather than more) rFD than young rats.

### Age-related amplification of rFD

rFD was greater in old as compared with young rat single fibres, and this finding aligns with our previous reports at the joint level in humans where rFD was greater during ankle dorsiflexion for older as compared with younger men (Power et al., 2014a). Additionally, in the present study, the magnitude of rFD was greater during the slow shortening speed condition as compared with fast, likely owing to the greater work of shortening performed in the slow condition (Fig. 4) (Fortuna et al., 2017; Maréchal and Plaghki, 1979; Meijer et al., 1998; Power et al., 2014a). rFD is believed to be proportional to work of shortening (i.e. the product of force and displacement), such that greater forces or shortening amplitudes increase the magnitude of rFD (Chen et al., 2020; Hahn et al., 2023; Herzog et al., 2000), probably as a result of actin angular deformation inhibiting cross-bridge attachment (Herzog et al., 2000; Josephson and Stokes, 1999; Joumaa et al., 2012, 2015). Instantaneous stiffness was decreased in both young and old fibres following active shortening as compared with the fixed-end isometric reference contractions, and this is consistent with previous findings that stiffness decreases in proportion to the magnitude of rFD, indicating a decreased proportion of attached cross-bridges (Lee and Herzog, 2003; Power et al., 2014a; Sugi and Tsuchiya, 1988). Our rFD values in both young and old fibres were tightly coupled with stiffness depression (%) (*R*^2^≈0.6–0.8; Fig. 5), which aligns with findings in young humans, rats and rabbits (Joumaa et al., 2012, 2015; Mashouri et al., 2021; Pinnell et al., 2019).

Single muscle fibres from old rats performed less mechanical work during active shortening (Fig. 4), and this is likely owing to age-related impairments in force production but also to a general slowing of maximal shortening velocity in old age (Hinks et al., 2024a; Power et al., 2013b; Thom et al., 2007). Therefore, the absolute shortening speeds used in the present study likely represented a relatively higher percentage of maximal shortening velocity in old as compared with young rats. Based on the force–velocity relationship, this further disadvantaged fibres from old rats for force production during a shortening contraction, ultimately resulting in an impaired ability to generate work (Joumaa et al., 2015). Interestingly, despite performing less mechanical work during the shortening contractions than young group, the old group experienced considerably greater rFD and stiffness depression (Fig. 4). Therefore, age-related changes to the structure and function of the contractile machinery likely altered this history-dependent property of muscle.

### Why did old incur a greater magnitude of rFD than young?

Based on the greater stiffness depression in old as compared with young rats (Fig. 4), there are fewer attached force producing cross-bridges during the isometric steady state following active shortening in old as compared with young rats. It is especially likely that greater stiffness depression contributed to the greater rFD in old age considering there were identical magnitudes of increase (38%) in both stiffness depression and rFD in old compared with young rats, with marked relationships between rFD and stiffness depression. Old rats, however, had weaker relationships between stiffness depression and rFD (*R*^2^=0.59–0.65) compared with young rats (*R*^2^=0.78–0.84), so other factors besides greater stiffness depression may have contributed to the age-related differences in single fibre rFD. For example, of the remaining attached cross-bridges in the rFD isometric steady state, there may be more cross-bridges in a weakly bound configuration in old as compared with young fibres (Frontera and Ochala, 2015; Ochala et al., 2007), which could enhance the magnitude of rFD. Further investigations of structural changes during and following active shortening are warranted to determine whether shortening-induced actin deformation is greater in old than in young fibres.

### rFE in old age

As expected, there was no difference in the magnitude of rFE between young and old rats. Furthermore, rFE was indeed greater on the descending limb than the plateau region of the force–length relationship (Edman et al., 1978; Rassier and Herzog, 2004) (Fig. 6).

rFE in older adult humans has previously been reported to be ∼2.5 times higher than in young adults for the ankle dorsiflexors (Power et al., 2012), while the knee extensors had a similar level of rFE compared with those of young adults, but the relative contribution of passive force to total force production was greater in the older adults (Power et al., 2013a). As well, the time to reach an isometric steady-state force level following active lengthening was reported to be longer in old when compared with young humans (Power et al., 2014b). Therefore, we have previously hypothesized that a passive structural mechanism within the sarcomere (i.e. titin) may play a disproportionately greater role in rFE in old compared with young humans (Power et al., 2012). However, given the absence of any age-related amplification of rFE at the cellular level in the present study (Fig. 6), we question our original working hypothesis. Therefore, greater rFE magnitudes in older adults at the joint level are likely driven by factors upstream of the cellular mechanisms of rFE. A plausible explanation is a greater relative excursion of muscle fascicles for a given joint angular rotation, owing to muscle architectural remodelling resulting in shorter fascicle lengths in old rats and humans (Hinks et al., 2024a; Narici et al., 2003; Power et al., 2013a, 2021). With shorter fascicles than those of young adults, muscles of older adults would be lengthened relatively more, and may fall further along the descending limb of the force–length relationship than those of young adults, which would be consistent with the finding that rFE is greatest following large stretch amplitudes to long muscle lengths (Bakenecker et al., 2022; Fukutani et al., 2017; Rassier and Herzog, 2004).

### Methodological considerations and future directions

It is important to note that this study investigated muscle fibres from male rats. Consequently, our results might differ if fibres from female rats had been tested, as female physiology and ageing – such as the hormonal changes during menopause (Hinks et al., 2024b; Hubbard et al., 2023; Mashouri et al., 2023) – could influence outcomes. Additionally, while previous studies found no sex differences in rFE at the joint level in young humans (Jacob et al., 2023; Jones et al., 2016), and despite variations in fibre type composition between males and females (Haizlip et al., 2015; Suzuki and Yamamuro, 1985), we observed no impact of fibre type on rFE magnitude in human vastus lateralis fibres (Pinnell et al., 2019). Although sex differences are likely minimal, a more detailed examination of how sex may affect the history dependence of force across different muscle scales is warranted. Additionally, the proximal portion of the psoas major is composed almost entirely of type II fibres. Therefore, our findings might not be applicable to muscles with a higher proportion of slow-type fibres. As noted above, the magnitude of rFE has been reported to be greater in slower type muscles, such as the soleus (Ramsey et al., 2010), though this is not always the case (Pinnell et al., 2019). Importantly, rFD is influenced by work, with fast-type muscles exhibiting higher rFD compared with slow-type muscles, unless the shortening speed is set relative to maximum shortening velocity (Joumaa et al., 2015). As ageing is associated with a shift towards a greater proportion of slow-type fibres (Larsson et al., 2019), the lack of fibre type assessment in our study might have contributed to variability between young and old rats. Although we did not find age-related differences in rFE in the psoas muscle of young and old rats, additional research scaling from single fibres to the joint level in humans is necessary to support or challenge previous findings (Power et al., 2012, 2014a, 2015). Furthermore, the experiments were conducted at 12°C, while *in vivo* muscle temperature is closer to 37°C. The magnitude of rFE (%) is greater at cooler (10°C) compared with warmer temperatures (35°C), as a result of the greater impairment in isometric force at lower temperatures (Roots and Ranatunga, 2008). Therefore, the role of temperature in the present study's findings on rFE scaling remains unclear and requires further investigation. Although the increased magnitude of rFE observed at the joint level in old as compared with young humans (Power et al., 2012, 2014a, 2015) does not seem to originate at the cellular level in rodents, the interaction between rFD and rFE during stretch–shortening cycles does enhance performance at the single fibre level (Patterson et al., 2024) and aligns with observations of improved joint-level power in humans (McDermott et al., 2023). Future research should consider these methodological factors when scaling these history-dependent properties from the cellular to joint level in humans.

Similar to previous single fibre studies (Mashouri et al., 2021; Pinnell et al., 2019; Tomalka et al., 2024), owing to the large heterogeneity in single muscle fibre mechanical function (factors: fibre type, sarcomere length non-uniformity, damage, ageing at different rates, etc.), we designed our study to treat each fibre as an independent sample in our statistical analysis, resulting in an unequal number of animals per group and fibres per animal. However, a nested statistical design with an equal number of animals per group and fibres per animal is often suggested to be a more rigorous approach (Wilkinson et al., 2023). Statistical analyses of data from single fibre experiments has been an ongoing challenge in this field, with no clear consensus on the optimal approach. Further research is needed comparing methods that treat fibres independently, average across fibres within a subject, and nest fibres within each subject to gain a better understanding of the best practices for designing experiments to optimize physiological relevance.

### Conclusion

We showed that rFD was amplified at the cellular level in old rat single muscle fibres owing to a greater inhibition of cross-bridge attachment as compared with young, while rFE was unaltered. While specific force (i.e. muscle quality) was not different between young and old fibres, there were underlying age-related modifications to the contractile machinery which led to alterations to rFD in old age. rFD and rFE are associated with poorer and better neuromuscular economy, respectively (de Brito Fontana et al., 2018; Jones et al., 2016; Joumaa and Herzog, 2013; Paquin and Power, 2018); thus, training interventions that decrease rFD and/or increase rFE could have the potential to improve neuromuscular economy (i.e. greater force for the same level of muscle activation). Improved neuromuscular economy could be beneficial for older individuals to improve function during everyday movements, which consist of both concentric (rFD) and eccentric (rFE) contractions.
